# An unusual high bifurcation and variable branching of the axillary artery in a Greek male cadaver

**DOI:** 10.1186/2193-1801-3-640

**Published:** 2014-10-28

**Authors:** Konstantinos Natsis, Maria Piagkou, Nikitas – Apollon Panagiotopoulos, Stylianos Apostolidis

**Affiliations:** Department of Anatomy, Medical School, Aristotle University of Thessaloniki, Post Box: 300, 54124 Thessaloniki, Greece; Department of Anatomy, Medical School, National and Kapodistrian University of Athens, M. Asias 75, 11527 Athens, Greece

**Keywords:** Axillary artery, Bifurcation, Superficial brachial artery, Anomaly, Variation

## Abstract

**Introduction:**

The axillary artery presents abnormalities in its origin and course and a variable branching.

**Case description:**

A rare case of axillary artery bifurcation and branching was observed in a 60-years-old European male cadaver of Greek origin. The right axillary artery at the second part was bifurcated into a superficial and a deep brachial artery. The superficial brachial artery anteromedial to the median nerve and lateral to the ulnar nerve gave off the acromio-thoracic artery and two lateral thoracic arteries. The deep brachial artery behind the median nerve, after giving rise to the anterior circumflex humeral artery trifurcated into a branch that coursed distally, the posterior circumflex humeral artery and the subscapular artery. The latter subdivided into the circumflex scapular artery, a muscular branch for the subscapularis and the thoracodorsal artery. The continuation of the deep brachial artery divided laterally into a humeral nutrient artery and medially into a trunk which trifurcated into the profunda brachii artery, a deep muscular branch and a branch to the posterior compartment of the arm. The profunda brachii artery ended as radial and middle collateral arteries.

**Discussion and evaluation:**

Deviations from the normal arterial pattern are of immense significance for anatomists, plastic, cardiovascular and orthopedic surgeons, vascular radiologists and interventional cardiologists.

## Introduction

The axillary artery (AA), a continuation of the subclavian artery extends from the lateral border of the first rib to the inferior border of the teres major muscle and then becomes the brachial artery. The pectoralis minor muscle divides the AA into three parts. The 1^st^ part gives rise to the superior thoracic artery (STA), the 2^nd^ part surrounded by the cords of the brachial plexus provides the acromio-thoracic (ATA) and the lateral thoracic arteries (LTAs) and the 3^rd^ part gives off the subscapular artery (SSA), the anterior and posterior circumflex humeral arteries (ACHA and PCHA) (Adachi 1928). Although this is the classic arterial pattern, branching abnormalities and rare anomalies in the AA origin and course may occur (Jurjus et al. 1999; Tan and Tan 1994). The variability of AA is of paramount importance for anatomists, plastic, cardiovascular and orthopedic surgeons, vascular radiologists and interventional cardiologists.

In the present case, a unilateral high bifurcation of the AA into a superficial brachial artery (SBA) in front of the median nerve (MN) and a deep brachial artery (DBA) behind the MN is described. This abnormal division of the artery may have clinical application in hemorrhagic emergencies, where the accurate diagnosis and surgical repair or ligation of the vessels is crucial.

## Case description

**Figure 1:**
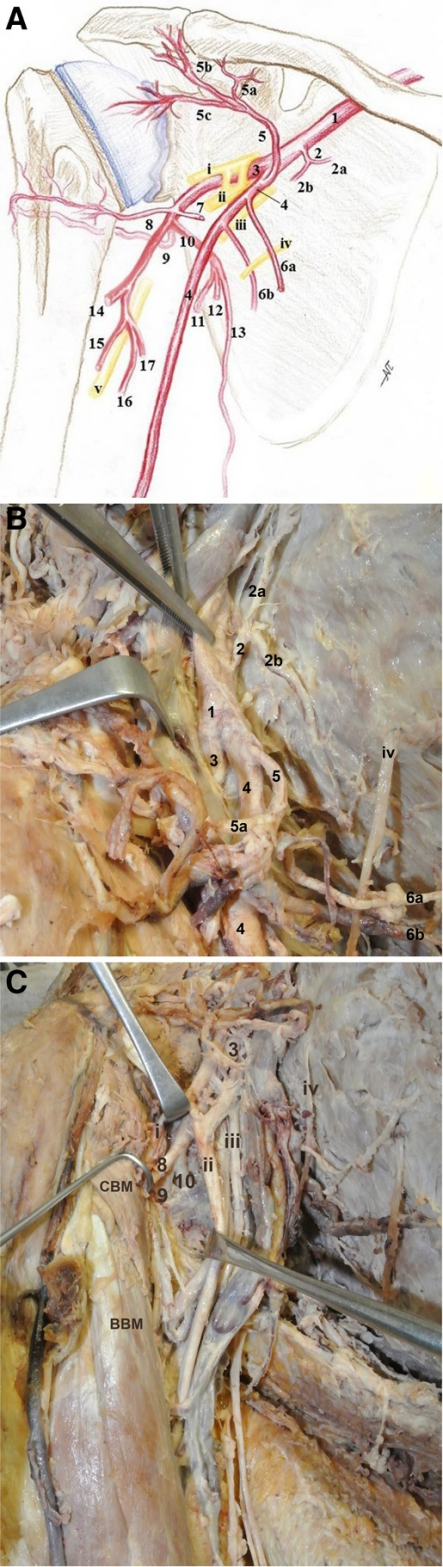
**The high bifurcation of the right axillary artery into a superficial and a deep brachial artery. (A)** Schematic drawing of the right axilla of a male cadaver, **1:** axillary artery, **2:** superior thoracic artery, **2a**, **2b:** 1^st^ and 2^nd^ branch of superior thoracic artery, **3:** deep brachial artery, **4:** superficial brachial artery, **5:** acromio-thoracic artery, **5a**, **5b**, **5c:** clavicular, deltoid and acromial branches of the acromio-thoracic artery, **6a**, **6b:** 1^st^ and 2^nd^ branch of the lateral thoracic artery, **7:** branch to subscapularis muscle, **8:** anterior circumflex humeral artery, **9:** posterior circumflex humeral artery, **10:** subscapular arterial trunk, **11:** circumflex scapular artery, **12:** branch for subscapularis muscle, **13:** thoracodorsal artery, **14:** radial collateral artery, **15:** artery accompanied the radial nerve in the spiral groove, **16:** deep muscular branch, **17:** branch to the posterior surface of the arm, **i:** musculocutaneous nerve, **ii:** median nerve, **iii:** ulnar nerve, **iv:** intercostobrachial nerve and **v:** radial nerve. **(B)** Photograph of the right axilla and the upper arm, **1:** axillary artery, **2:** superior thoracic artery, **2a**, **2b:** 1^st^ and 2^nd^ branch of the superior thoracic artery, **3:** deep brachial artery, **4:** superficial brachial artery, **5:** acromio-thoracic artery, **5a**, **5b**, **5c:** clavicular, deltoid and acromial branches of the acromio-thoracic artery, **6a**, **6b:** 1^st^ and 2^nd^ branch of the lateral thoracic artery. **(C)** Photograph of the right axilla and the upper arm, **3:** deep brachial artery, **8:** anterior circumflex humeral artery, **9:** posterior circumflex humeral artery, **10:** subscapular arterial trunk, **i:** musculocutaneous nerve, **ii:** median nerve, **iii:** ulnar nerve, **iv:** intercostobrachial nerve and **v:** radial nerve, **CBM:** coracobrachialis muscle, **BBM:** biceps brachialis muscle.

**Figure 2:**
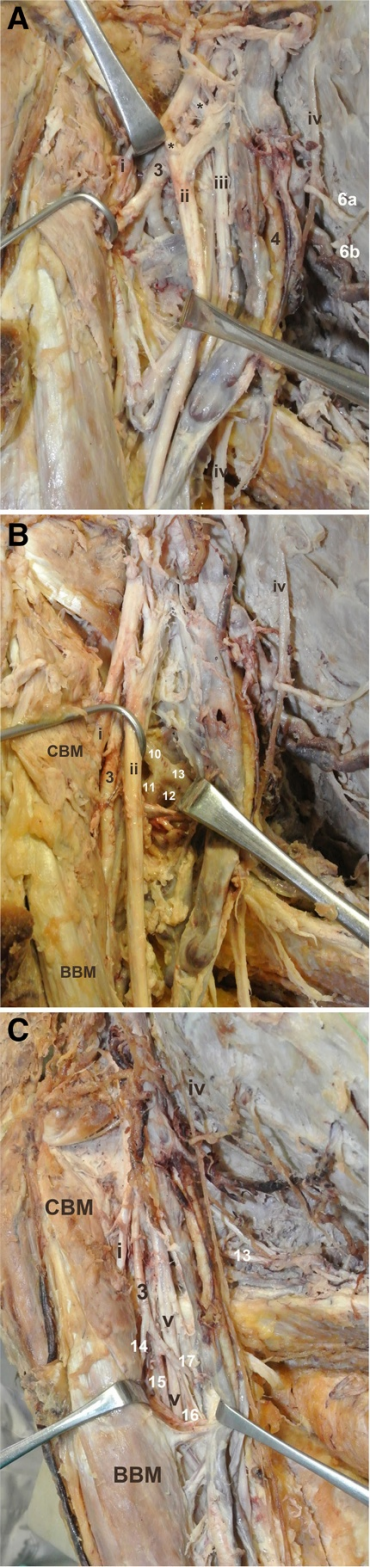
**The variable branching pattern of the superficial and the deep brachial arteries.** **(A)** Photograph of the right axilla and the upper arm, **3:** deep brachial artery, **4:** superficial brachial artery, **6a**, **6b:** 1^st^ and 2^nd^ branch of the lateral thoracic artery, **i:** musculocutaneous nerve, **ii:** median nerve, **iii:** ulnar nerve, **iv:** intercostobrachial nerve and **v:** radial nerve, **: double anastomosis. **(B)** Photograph of the right axilla and the upper arm, **3:** deep brachial artery, **10:** subscapular arterial trunk, **11:** circumflex scapular artery, **12:** branch for subscapularis muscle, **13:** thoracodorsal artery, **i:** musculocutaneous nerve, **ii:** median nerve, **iv:** intercostobrachial nerve and **v:** radial nerve, **CBM:** coracobrachialis muscle, **BBM:** biceps brachialis muscle. **(C)** Photograph of the right axilla and the upper arm, **3:** deep brachial artery, **13:** thoracodorsal artery, **14:** radial collateral artery, **15:** artery accompanied the radial nerve in the spiral groove, **16:** deep muscular branch, **17:** branch to the posterior surface of the arm, **i:** musculocutaneous nerve, **v:** radial nerve, **CBM:** coracobrachialis muscle, **BBM:** biceps brachialis muscle.

During routine dissection of a 60-years-old Greek male donor who died after cardiac arrest, an unusual case of high bifurcation of the AA into two arterial stems supplying the right axilla and the upper limb was observed (Figure 1A). The project that conforms to the provisions of the Helsinki Declaration of 1995 (as revised in Edinburgh 2000) has been approved by the Ethics Committee of the Medical School of the Aristotle University of Thessaloniki. The body donor gave written informed consent and his anonymity has been preserved.The AA (diameter 1 cm), at the lateral border of the first rib gave off the STA that bifurcated behind the pectoralis minor muscle (Figure 1B). Deeply to the medial margin of the pectoralis minor, at a distance 1.4 cm distal to the STA origin and 0.9 cm proximal to the ATA origin, the AA bifurcated into the SBA and DBA (diameters 7 and 8 mm). The SBA courses anteromedially to the MN and laterally to the ulnar nerve and the basilic vein and the DBA was located laterally to the MN, deep to the fork of the MN roots that formed a double anastomosis (Figure 2A). The complex of the ATA divided into clavicular, pectoral, deltoid and acromial branches of which the pectoral branch was the largest. At distances 2.8 and 4.3 cm distal to the AA bifurcation, the SBA gave off two LTAs, through which the intercostobrachial nerve passed to the arm (Figure 1B). The SBA behind the bicipital aponeurosis bifurcated into radial and ulnar arteries. The DBA, at distances 4.5 and 5 cm distal to the AA bifurcation, after giving off a muscular subscapular branch and the ACHA, trifurcated into the continuation of the DBA, the PCHA and the SSA (Figure 1C). The ACHA passed deep to the coracobrachialis and biceps brachii muscles, toward the surgical neck of the humerus and the PCHA accompanied the axillary nerve in the quandrangular space. The SSA divided into the circumflex scapular artery (CSA), a branch for the subscapularis and the thoracodorsal artery (TDA) (Figure 2B). Finally, the distal part of the DBA (9.4 cm below the AA bifurcation) gave off a humeral nutrient artery and a short trunk that further subdivided into the profunda brachial artery (PBA) which accompanied the radial nerve in the spiral groove, a deep muscular branch and a branch to the posterior compartment of the arm. The PBA ended as the radial and middle collateral arteries (Figure 2C). However, such variation was absent on the left side.

## Discussion and evaluation

The coexistence of abnormalities of the AA (variable origin, abnormal course and aberrant or variable branches) is unusual, while isolated aberrations of the arterial pattern follow a wide range among races (Yang et al. 2008). Many theories on the embryologic development of the upper limb arteries have been proposed and the last decade, Rodríguez-Niedenführ et al. (2001) after a detailed embryological study concluded that the arterial system of the upper limb develops by selective enlargement or regression of a capillary plexus and not by budding from a main axial trunk and this development is closely related to the bone development. The arterial variations can be explained as a deviation from the normal vascular pattern (Konarik et al. 2009; Singer, 1933) and especially the SBA presence is based on the persistence of more than one intersegmental cervical artery, which remains and can even increase in size (Jurjus et al. 1999).

Twenty-three different arterial patterns of the AA have been recognized (De Garis and Swartley 1928) and female predilection is reported (Pandey et al. 2004; Trotter et al. 1930). The AA may give off 5–11 branches, the commonest number being 8 (De Garis and Swartley 1928). The STA, the LTA and branches of the 3^rd^ part are extremely variable, as opposed to the constant ATA. In order of frequency, the STA may arise from the 1^st^ part of the AA, the ATA, the subclavian artery, the 2^nd^ part of the AA, or it may be absent, or it may arise from the LTA (Huelke 1959). The LTA may arise from the 1^st^ part of the AA (Patnaik 2000) or from the TDA (Saadeh 1984). The TDA may arise from the LTA or by a common trunk with the LTA, CSA and both the circumflex humeral arteries. Occasionally, the TDA may be double, as in our case, or triple (Patnaik 2000). Moreover, the AA may give off accessory TDAs unilaterally (1^st^ part) and/or bilaterally (3^rd^ part) (Natsis et al. 2006b; Saadeh 1984). The ACHA and PCHA, as well as the PCHA and SSA (Agrawal et al. 2013) may derive from a common trunk (Adachi 1928). The PCHA may originate from the SSA (Keen 1961). A high origin of the SSA from the 1^st^ part and the 2^nd^ part of the AA was also reported (Huelke 1959). In our case, the DBA after giving off the ACHA trifurcated into the PCHA, the SSA and a branch that continued in the arm. The SSA originated as normal, 4.1 cm below the ATA, contrariwise to Keen (1961) who observed a high origin in 28.9%. The ACHA, the PCHA, the SSA, the radial collateral, middle collateral and superior ulnar collateral arteries may emerge from the 3^rd^ part of the AA, although the PBA may be absent (Samuel et al. 2006). The extremely variable PBA, following the radial nerve, may be the terminal part of the AA, or arise from the PCHA or arise by a common trunk with the PCHA and the SSA (Keen 1961).

The AA high bifurcation into SBA and DBA (Cavdar et al. 2000; Yotova and Novakov 2004) coursing parallel is more frequent in African-Americans (13.4%) than in Caucasians (4.6%) (De Garis and Swartley 1928). Trotter et al. (1930) found no racial differences. A unique case of AA trifurcation into SBA, PBA and SSA was also reported (Kachlik et al. 2011). The SBA, especially the height of its emersion from the AA gathers the greatest interest. Recently, Kachlik et al. (2011), Patnaik (2000) and Yang et al. (2008) mentioned the unilateral presence of the SBA in 5–12.2%, while Jurjus et al. (1999) and Yang et al. (2008) described sporadic cases of bilateral occurrence. The presence of the SBA is more frequent in males and on the right side (Rodriguez-Niedenfuhr et al. 2001). In cases in which the SBA gave no branches (Cavdar et al. 2000), the DBA supplied the whole area (VijayaBhaskar et al. 2006). In our case, similarly to Maraspin (1971), Rao et al. (2012) and Yotova & Novakov (2004), a high division of the 2^nd^ part of the AA into SBA and DBA occurred. Other authors (Cavdar et al. 2000; Desai et al. 2011; Kachlik et al. 2011; Patnaik et al. 2001; VijayaBhaskar et al. 2006) found the AA bifurcation at the 3^rd^ part, while a rare bifurcation at the 1^st^ part, absence of the SSA and origin of the ATA from the DBA, was also referred (Jurjus et al. 1999). In our case, unlike to Jurjus et al. (1999), the DBA was larger than the SBA and the latter bifurcated into radial and ulnar arteries as usual, while in other studies, the SBA ended in the arm or continued as radial (high origin of radial) (Cavdar et al. 2000; Keen 1961; Natsis et al. 2009; Rodriguez-Niedenfuhr et al. 2001) or ulnar artery (high origin of ulnar) with both arteries coursing superficially (Keen 1961; Natsis et al. 2006a; Rodriguez-Niedenfuhr et al. 2001). Jayakumari et al. (2006) described a SBA divided into radial and common interosseous arteries. On the other hand, in our case the DBA descended dorsally to the MN, giving off the nutrient humeral artery and a common trunk for the PBA, a deep muscular branch and a branch to the posterior compartment of the arm.

The variable pattern of the AA is of paramount importance for surgeons and interventional physicians. Although the superficial course of the SBA makes the arterial grafting and cardiac catheterization easier, the high bifurcation of the AA and its abnormal branching pattern may pose problems to clinician during angiographic procedures leading to diagnostic errors. The SBA due to its abnormal origin and position may be more prone to serious injury leading to hemorrhage (Jurjus et al. 1999) or to pseudoaneurysm (Yagain et al. 2012). The possibility to be mistaken for a vein is evident; leading to accidental intra-arterial injection and as a consequence the thrombosis or gangrene (Cohen, 1948; Pandey et al. 2004). During axillary approach, the transverse incision in dislocated shoulders may jeopardize the abnormal AA branches. Therefore, the variable arterial pattern is important to be identified preoperatively using Doppler ultrasound imaging or angiography, especially in emergency cases of chest wall reconstruction such as in Poland’s Syndrome (Shipkov et al. 2000) or during breast cancer surgery and axillary lymph nodes dissection, when surgeons have to correctly identify and protect the axillary vessels (Jurjus et al. 1999).

## Conclusions

The present case provides additional information on the branching and distribution pattern of the AA since the high division of the artery occurs at the 2^nd^ part. Documentation of such rare abnormalities in the axilla is highly significant for aneurysms and trauma surgery (Ortiz-Pomales et al. 2014) and angiography, where all therapeutic gestures should be performed with accuracy due to the possibility of iatrogenic injury, amputation of the arm or the fingers and further medico-legal implications.

## Consent

The body donor gave written informed consent before its death for the publication of this case report.

## Authors’ details

K. Natsis - Professor, Director of the Department of Anatomy, Medical School, Aristotle University of Thessaloniki, Greece, natsis@med.auth.gr

M. Piagkou - Assistant Professor, Department of Anatomy, Medical School, National and Kapodistrian University of Athens, Greece, mapian@med.uoa.gr

NA. Panagiotopoulos - Research Fellow, Department of Anatomy, Medical School, National and Kapodistrian University of Athens, Greece, panagiotopoulos.nikitas@gmail.com

S. Apostolidis - Associate Professor, Department of Anatomy, Medical School, Aristotle University of Thessaloniki, Greece, stlsa@med.auth.gr

## Abbreviations

AA: Axillary artery
STA: Superior thoracic artery
ATA: Acromio-thoracic artery
LTA: Lateral thoracic artery
SSA: Subscapular artery
ACHA: Anterior circumflex humeral artery
PCHA: Posterior circumflex humeral artery
SBA: Superficial brachial artery
MN: Median nerve
DBA: Deep brachial artery
CSA: Circumflex scapular artery
TDA: Thoracodorsal artery
PBA: Profunda brachial artery.
